# Optimal Population of FoxP3^+^ T Cells in Tumors Requires an Antigen Priming-Dependent Trafficking Receptor Switch

**DOI:** 10.1371/journal.pone.0030793

**Published:** 2012-01-23

**Authors:** Chuanwu Wang, Jee H. Lee, Chang H. Kim

**Affiliations:** Laboratory of Immunology and Hematopoiesis, Department of Comparative Pathobiology, Purdue Cancer Center, Bindley Bioscience Center, Purdue University, West Lafayette, Indiana, United States of America; University of Palermo, Italy

## Abstract

FoxP3^+^ T cells populate tumors and regulate anti-tumor immunity. The requirement for optimal population of FoxP3^+^ regulatory T cells in tumors remains unclear. We investigated the migration requirement and stability of tumor-associated FoxP3^+^ T cells. We found that only memory, but not naïve, FoxP3^+^ T cells are highly enriched in tumors. Almost all of the tumor-infiltrating FoxP3^+^ T cells express Helios, an antigen associated either with thymus-generated FoxP3^+^ T cells or activated T cells in the periphery. The tumor-infiltrating FoxP3^+^ T cells largely lack CD62L and CCR7, two trafficking receptors required for T cell migration into secondary lymphoid tissues. Instead, the tumor infiltrating FoxP3^+^ T cells highly express memory/tumor-associated CCR8 and CXCR4. Antigen priming is required for induction of this trafficking receptor phenotype in FoxP3^+^ T cells and only antigen primed, but not antigen-inexperienced naive, FoxP3^+^ T cells can efficiently migrate into tumors. While the migration of FoxP3^+^ T cells into tumors was a readily detectable event, generation of induced FoxP3^+^ T cells within tumors was unexpectedly inefficient. Genetic marking of current and ex-FoxP3^+^ T cells revealed that tumor-infiltrating FoxP3^+^ T cells are highly stable and do not readily convert back to FoxP3^−^ T cells. Taken together, our results indicate that population of tumors with thymus-generated FoxP3^+^ T cells requires an antigen priming-dependent trafficking receptor switch in lymphoid tissues.

## Introduction

FoxP3^+^ regulatory T cells (Tregs) play critical roles in inducing tolerance in the body [1]–[6]. Naïve FoxP3^+^ T cells are generated in the thymus and programmed to migrate mainly to secondary lymphoid tissues [7]. Memory FoxP3^+^ T cells are generated from the thymus-generated naïve FoxP3^+^ T cells or naïve FoxP3^−^ T cells in secondary lymphoid tissues following antigen priming. Memory FoxP3^+^ T cells are heterogeneous in trafficking potentials. Some memory FoxP3^+^ T cells migrate into various non-lymphoid tissues, whereas others preferentially migrate into secondary lymphoid tissues [7].

FoxP3^+^ T cells can suppress anti-tumor immunity, and depletion of these cells using anti-CD25 antibody or cyclophosphamide was effective in eradicating some transplantable tumors in animals [8]–[12]. Depletion of Tregs led also to slower and inefficient induction of tumors by carcinogens such as methylcholanthrene [13]. FoxP3^+^ T cells suppress the anti-tumor effector function of both CD4^+^ Th1 cells and cytolytic CD8^+^ T cells [14], [15]. For some tumors, inflammation can promote tumorigenesis. For these types of tumors, FoxP3^+^ T cells could affect tumorigenesis by decreasing inflammation. FoxP3^+^ T cells are frequently found also in human tumors, and their presence within some types of tumors predicts reduced patient survival [16]–[18]. In other tumor types, however, the number of FoxP3^+^ T cells positively correlated with the patient survival rate [19]–[22]. For some tumors, no correlation between tumor-infiltrating FoxP3^+^ T cells and the patient survival rate was observed. Thus, the roles of tumor-associated FoxP3^+^ T cells appear to be complex.

The requirement for population of FoxP3^+^ T cells in tumors is incompletely understood. To account for the enrichment of Tregs in tumors, several explanations have been provided. For example, migration of Tregs into tumors in response to certain chemokines has been proposed [16], [23], [24]. Induction of FoxP3^+^ Tregs in tumors from conventional T cells in response to dendritic cells has been proposed as well [25]–[28]. Another explanation comes from the evidence that Tregs undergo proliferation with a low rate of apoptosis in tumors [29], [30]. Despite these explanations, many questions remain unanswered. 1) Are tumor-infiltrating FoxP3^+^ T cells the result of conversion from naïve FoxP3^−^ T cells in tumors or migration of pre-existing FoxP3^+^ T cells? 2) What is the requirement for FoxP3^+^ T cells to migrate into tumors? 3) Are the FoxP3^+^ T cells in tumors stable or do they become conventional FoxP3^−^ T cells in tumors? To provide more insights into the biology of FoxP3^+^ T cells in tumors, we studied the phenotype, induction, stability, and migration behavior of FoxP3^+^ T cells in tumors. Our results indicate that the antigen priming-dependent switch of trafficking receptor phenotype is important for infiltration of FoxP3^+^ T cells in tumors.

## Methods

### Mice, cell isolation, and tumor cell lines

BALB/c mice and C57BL/6 mice (6 to 8 weeks old) were purchased from Harlan (Indianapolis, IN). FoxP3-GFP-Cre×Rosa-tdTomato mice [31], CCR7 (−/−) mice, and B6.Cg-Foxp3^sf^/J mice were purchased from the Jackson laboratory. Rat insulin promoter (RIP)-membrane-bound ovalbumin (mOVA)×DO11.10 Treg mice in Rag2 (−/−) background were described before [7]. DO11.10 TCR Rag2 (−/−) transgenic mice were purchased from Taconic Farms. All animals were used according to the protocols (04-048 and 02-067) approved by the Purdue Animal Care and Use Committee. A20, B16, CT26 and 4T1 tumor cell lines were purchased from American Type Culture Collection (ATCC, Rockville, MD, USA). For ovalbumin (OVA) expressing tumor cells, A20 cells, and 4T1 cells were transfected with linearized pAC-neo-OVA [32] or pCDNA3.1-hygromycin-OVA by using Gene Pulser (at 250 V, 975 µF; Bio-Rad, Hercules, CA, USA). Stably transfected cells were selected with G418 (1 mg/ml for pAC-neo-OVA) or hygromycin B (0.4 mg/ml for pCDNA3.1-Hygromycin) for 2–3 weeks. CD4^+^ T cells (purity >95%) were isolated as described before [33].

### Tumor formation in mice

A20 (or A20-OVA), CT26, and 4T1 (or 4T1-OVA) cells were cultured in RPMI 1640 (Invitrogen) supplemented with 10% fetal bovine serum (FBS). B16 cells were cultured in Dulbecco's modified Eagle medium (DMEM) supplemented with 10% FBS. A20 cells (2×10^6^ cells/mouse) or CT26 cells (3×10^5^ cells/mouse) were injected s.c. in the right flank of BALB/c mice. B16 cells (2×10^5^ cells/mouse) were similarly injected into C57BL/6 mice. 4T1 cells (1×10^4^ cells/mouse) were implanted s.c. in the mammary glands of female BALB/c mice. Tumor size was measured daily with a digital caliper. Most experiments were performed when the tumor diameter was ∼10 mm.

### Assessment of expression of trafficking receptors and Helios

Single-cell suspensions of tumor, draining inguinal lymph node (D-LN), or mixed peripheral lymph nodes (inguinal, auxiliary, and brachial) were prepared by grinding tissues through an iron mesh. The cells were stained with an antibody to CCR7 (clone 4B12; eBioscience) or CXCR4, (2B11; BD Biosciences) or rat control IgG2a antibody (clone RTK2758; BioLegend) for 30 min on ice. The cells were additionally stained with biotinylated anti-rat IgG2a (RG7/1.30; BD Biosciences) for 30 min, followed by staining with allophycocyanin-streptavidin (BD Biosciences) and antibodies to CD62L (clone MEL-14; BioLegend), CD44 (IM7; BioLegend), and CD4 (clone RM4-5; BioLegend). The cells were further stained with antibodies to FoxP3 (FJK-16s; eBioscience) and/or Helios (22F6; BioLegend) according to the manufacturer's protocol. CCR8 expression was detected with a home-made mCCL1-hFc protein, biotin-anti-hIgG (Vector Lab) and allophycocyanin-streptavidin.

### Homing experiments

For homing experiments, splenocytes and lymph node cells isolated from RIP-mOVA×DO11.10 Treg-enriched mice were prepared and cultured in the presence of hIL-2 (25 U/ml; PeproTech, Rocky Hill, NJ) and the peptide OVA_323–339_ (1 µg/ml) for 5 days. Activated KJ1-26^+^ FoxP3^+^ T cells (∼1×10^7^ cells/mouse) were labeled with carboxyfluorescein diacetate succinimidyl ester (CFDA-SE) and injected via a tail vein of mice when tumors reach a diameter of ∼10 mm. The KJ1-26^+^ FoxP3^+^ cells that migrated into various tissue sites were identified with flow cytometry using antibodies to DO11.10 TCR (KJ1-26) and FoxP3. Similar homing experiments were performed with wild type and CCR7 (−/−) CD4^+^ cells isolated from spleen and lymph nodes (inguinal, auxiliary, and brachial) and antigen-primed in vitro in complete RPMI 1640 medium (10% FBS) supplemented with concanavalin A (0.5 µg/ml, Sigma) and hIL-2 for 5 days. Wild type cells (1×10^7^ cells/mouse; labeled with tetramethylrhodamine isothiocyanate (TRITC)) and CCR7 (−/−) cells (1×10^7^ cells/mouse; labeled with CFDA-SE) were co-injected i.v. into C57BL/6 mice each bearing a tumor of ∼10 mm diameter. The host mice were sacrificed 36 h later, and indicated organs were harvested. The numbers of injected CFDA-SE^+^ or TRITC^+^ FoxP3^+^ cells migrated into each organ were determined with flow cytometry. Absolute numbers of T cells that migrated into various organs were determined as described before [7]. We also normalized the absolute number of the T cells present in each organ with that of the injected cells to obtain a parameter termed ‘homing index.’

### Induction of FoxP3^+^ T cells in lymph nodes versus tumors

Splenocytes isolated from DO11.10 Rag2 (−/−) mice (2×10^6^ cells/mouse; containing naïve FoxP3^−^ but not FoxP3^+^ CD4^+^ T cells) were injected i.v. into Rag2 (−/−) BALB/c mice, and then the mice were implanted with A20-OVA tumor cells 15 hours later. Mice were sacrificed 2 weeks later when tumor size was ∼10 mm, and conversion rate of naïve FoxP3^−^ CD4^+^ T cells into FoxP3^+^ T cells (represented by % FoxP3^+^ cells of KJ1.26^+^ CD4^+^ T cells) was determined in various organs and tumors.

### Comparison of the population of wild type and CCR7 (−/−) FoxP3^+^ cells in tumors

Wild type CD45.1^+^ CD4^+^ T cells and CCR7 (−/−) CD45.2^+^ CD4^+^ T cells (containing ∼2×10^6^ FoxP3^+^ cells/mouse) were intraperitoneally transferred into scurfy mice deficient with FoxP3 expression on day 2 following birth. At 5 weeks of age, B16 tumor cells were implanted into the flank of scurfy mice that were previously co-injected with wild type and CCR7 (−/−) CD4^+^ T cells. When the tumor size was ∼10 mm, the indicated organs were harvested, and the frequency and phenotype of FoxP3^+^ cells were determined with flow cytometry. In separate experiments, wild type and CCR7 (−/−) CD4^+^ T cells were separately injected into scurfy mice for assessment of their population in tumors in a non-competitive setting.

### Chemotaxis

Chemotaxis was assessed using Transwell chambers (5 µm pores; Corning). Total lymph node cells and B220^+^ A20 tumor cell-depleted tumor mononuclear cells were used for the chemotaxis assay. 0.5 million cells were added to the top chambers and chemokines were added at optimal concentrations (1000 ng/ml of CCL1; 500 ng/ml of CCL19; 1000 ng/ml of CCL21; 250 ng/ml of CXCL12; all are mouse chemokines purchased from R&D systems or PeproTech) in the lower chambers. Cells were allowed to migrate for 3 h. The cells migrated to the lower chambers were stained with antibodies to CD4 and FoxP3 and analyzed by flow cytometry as described previously [34]. The numbers of migrated cells were normalized for the numbers of the same cell types in input cells to obtain the index “% migration.”

### Confocal microscopy

A20 tumors were harvested from BALB/C mice when the tumors reach ∼10 mm in diameter. Frozen sections (8 µm) of A20 tumors were stained with antibodies to CD4, FoxP3 and Helios for 40 minutes as described previously [33]. The images were acquired with a Leica SP5 II confocal microscope housed in the Super-resolution Imaging Lab (Purdue Veterinary Medicine).

### Statistical analyses

Averages with SEM are shown in most of the figures. Student's paired t test (2-tailed) or Mann-Whitey test was used to determine the significance of differences between two groups. p values<or = 0.05 were considered significant.

## Results

### Tumors are enriched with Helios^+^ FoxP3^+^ T cells

**Figure 1:**
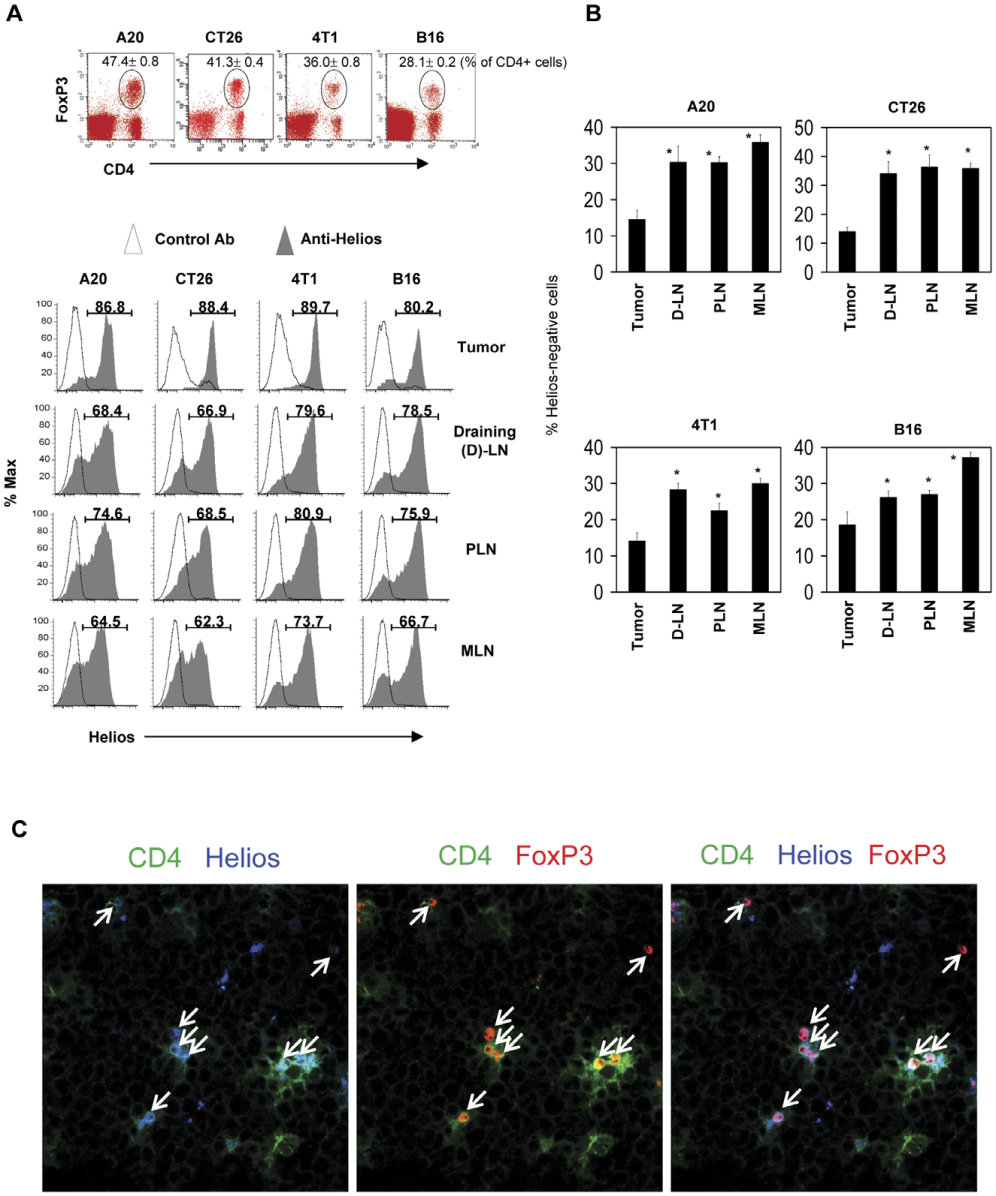
Tumor-infiltrating FoxP3^+^ T cells express the Helios antigen. Mice harboring one of the four types of tumors (A20 B cell lymphoma, CT26 colon cancer, 4T1 breast cancer, and B16 melanoma) were examined for expression of Helios by FoxP3^+^ T cells. (A) Helios expression by FoxP3^+^ T cells in tumors and various secondary lymphoid tissues such as the draining lymph node (D-LN), other peripheral lymph nodes (PLN), and mesenteric lymph node (MLN). (B) Frequencies of Helios-negative FoxP3^+^ T cells in tumors and secondary lymphoid tissues. (C) Detection of Helios^+^ FoxP3^+^ T cells in tumors by confocal microscopy. Arrows indicate T cells that co-express FoxP3 and Helios in tumors. Representative and combined (n = 4) data are shown. “*” indicates significant differences from tumors.

Helios is known to be preferentially expressed by thymus-derived FoxP3^+^ T cells and activated T cells in proliferation [35], [36]. To gain insights into the phenotype of FoxP3^+^ T cells populating tumors, we examined the expression of Helios in FoxP3^+^ T cells infiltrating tumors formed in mice. Interestingly, frequencies of Helios-expressing FoxP3^+^ T cells were highest in tumors among the tissues examined, which included the draining lymph node (D-LN), non-draining peripheral lymph nodes (PLN), and mesenteric lymph nodes (MLN) (Fig. 1A and B). We examined four different tumor types (A20 B cell lymphoma, CT26 colon cancer, 4T1 breast cancer, and B16 melanoma), and the results were comparable with all of the tumor types. Furthermore, we were able to observe the presence of these Helios^+^ FoxP3^+^ T cells infiltrating A20 tumors using an immunohistochemistry technique (Fig. 1C).

### Induced FoxP3^+^ T cells are found more frequently in tumor-draining lymph nodes than in tumors

**Figure 2:**
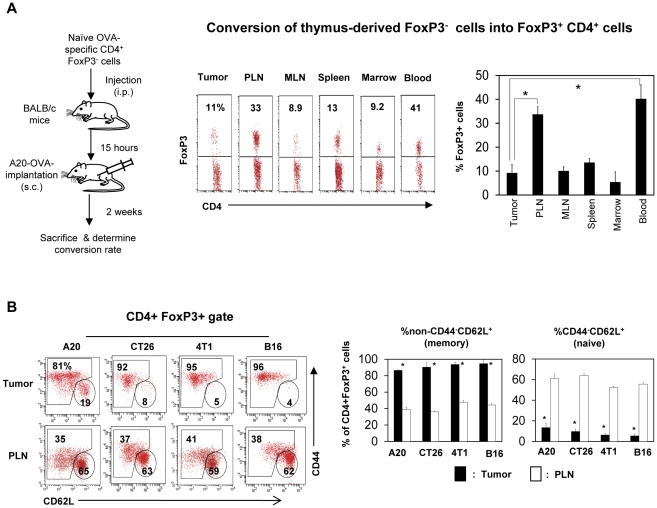
Induction of FoxP3^+^ regulatory T cells in tumors is inefficient, and most tumor-infiltrating FoxP3^+^ T cells have the memory phenotype. (A) Splenocytes of DO11.10 Rag2−/− mice (containing naïve FoxP3^−^ CD4^+^ T cells but not FoxP3^+^ T cells) were injected i.v. into BALB/c mice, and A20 tumor cells expressing OVA were implanted 15 hours later. Mice were sacrificed 2 weeks later, and the conversion rate of naïve FoxP3^−^ CD4^+^ T cells into FoxP3^+^ T cells (represented by % FoxP3^+^ of KJ-1.26^+^ CD4^+^ T cells) was determined in various organs and tumors. (B) Memory FoxP3^+^ T cells are enriched in tumors. Expression of CD44 and CD62L by FoxP3^+^ T cells in tumors and peripheral lymph nodes were compared. Representative and combined data obtained from 3 (A) or 5–8 different experiments (B) are shown. “*” indicates significant differences between the tumor and indicated organs (A) or PLN (B).

A potential way to increase the number of tumor-infiltrating FoxP3^+^ T cells is through efficient conversion of FoxP3^−^ T cells into FoxP3^+^ T cells in tumors. We examined this possibility utilizing DO11.10 Rag2 (−/−) T cells, which express TCR specific for an OVA epitope. Almost all T cells in DO11.10 Rag2 (−/−) mice are FoxP3^−^ T cells because thymus-derived FoxP3^+^ T cells are not made in these mice due to Rag2 deficiency. We injected DO11.10 Rag2 (−/−) FoxP3^−^ T cells into A20-OVA tumor-bearing mice and determined the frequency of FoxP3^+^ T cells converted from FoxP3^−^ T cells 2 weeks after transfer of the cells. We found that peripheral lymph nodes and the blood were the compartments that had the highest frequencies (∼35%) of OVA-specific FoxP3^+^ T cells (Fig. 2A). In contrast, the frequency in tumors was only ∼10%, which is the basal level similar to that of other organs such as MLN, spleen and marrow. The data suggest that the conversion is more active in peripheral lymph nodes than in tumors.

### Tumor-infiltrating FoxP3^+^ T cells are largely composed of memory type cells

FoxP3^+^ T cells, generated in the thymus, are similar to conventional naïve CD4^+^ T cells in trafficking potential [37]. They are identified as CD44^−^CD62L^+^ cells. Upon antigen priming in secondary lymphoid tissues such as draining lymph nodes, thymus-derived FoxP3^+^ T cells lose CD62L but up-regulate CD44. Interestingly, the majority of FoxP3^+^ T cells in tumors expressed CD44 but not CD62L, which is a memory T cell phenotype (Fig. 2B). In contrast, the majority of FoxP3^+^ T cells in lymph nodes had the naïve cell phenotype.

### Tumor-infiltrating FoxP3^+^ T cells lack secondary lymphoid tissue homing receptors

**Figure 3:**
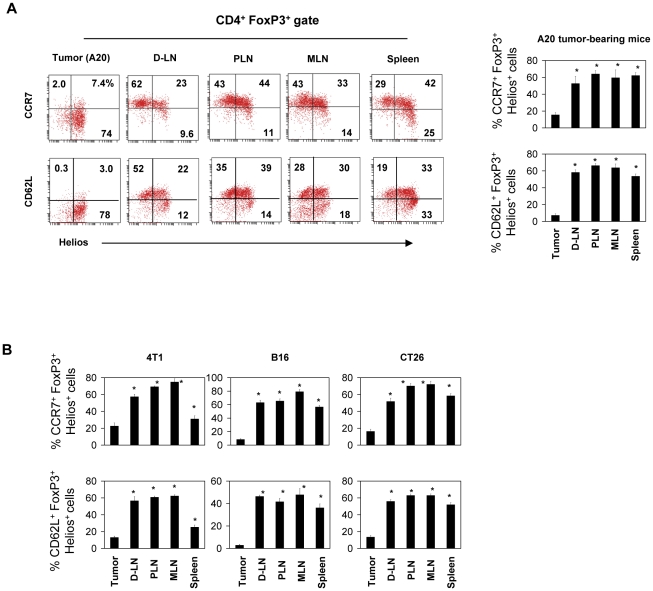
Tumor-infiltrating Helios^+^ FoxP3^+^ T cells are down-regulated for CD62L and CCR7. (A) Expression of CCR7 and CD62L by Helios^+^ FoxP3^+^ T cells in indicated organs and tumors (A20). (B) Expression of CCR7 and CD62L by Helios^+^ FoxP3^+^ T cells in indicated tumors and organs. Representative and combined data obtained from 4–6 different experiments are shown. “*” indicates significant differences from the tumor.

All naïve type FoxP3^+^ T cells highly express CD62L and CCR7, two major trafficking receptors for migration into peripheral lymph nodes [5]. Memory type FoxP3^+^ T cells are divided into CCR7^+^ secondary lymphoid tissue-homing cells and CCR7^−^ non-lymphoid tissue-homing cells. To determine which memory cell type is populating tumors, we examined expression of CCR7 by the FoxP3^+^ T cells in tumors (Fig. 3A). Helios^+^ FoxP3^+^ T cells in A20 B cell lymphoma tumors largely lacked CCR7 expression. At the same time, these FoxP3^+^ T cells in tumors lacked CD62L expression. In contrast, draining peripheral lymph nodes and other lymphoid tissues had many FoxP3^+^ T cells expressing CCR7 and CD62L. The FoxP3^+^ T cells in other tumor types were similarly deficient in expression of CCR7 and CD62L (Fig. 3B).

### Active migration of memory, but not naïve, FoxP3^+^ T cells into tumors

**Figure 4:**
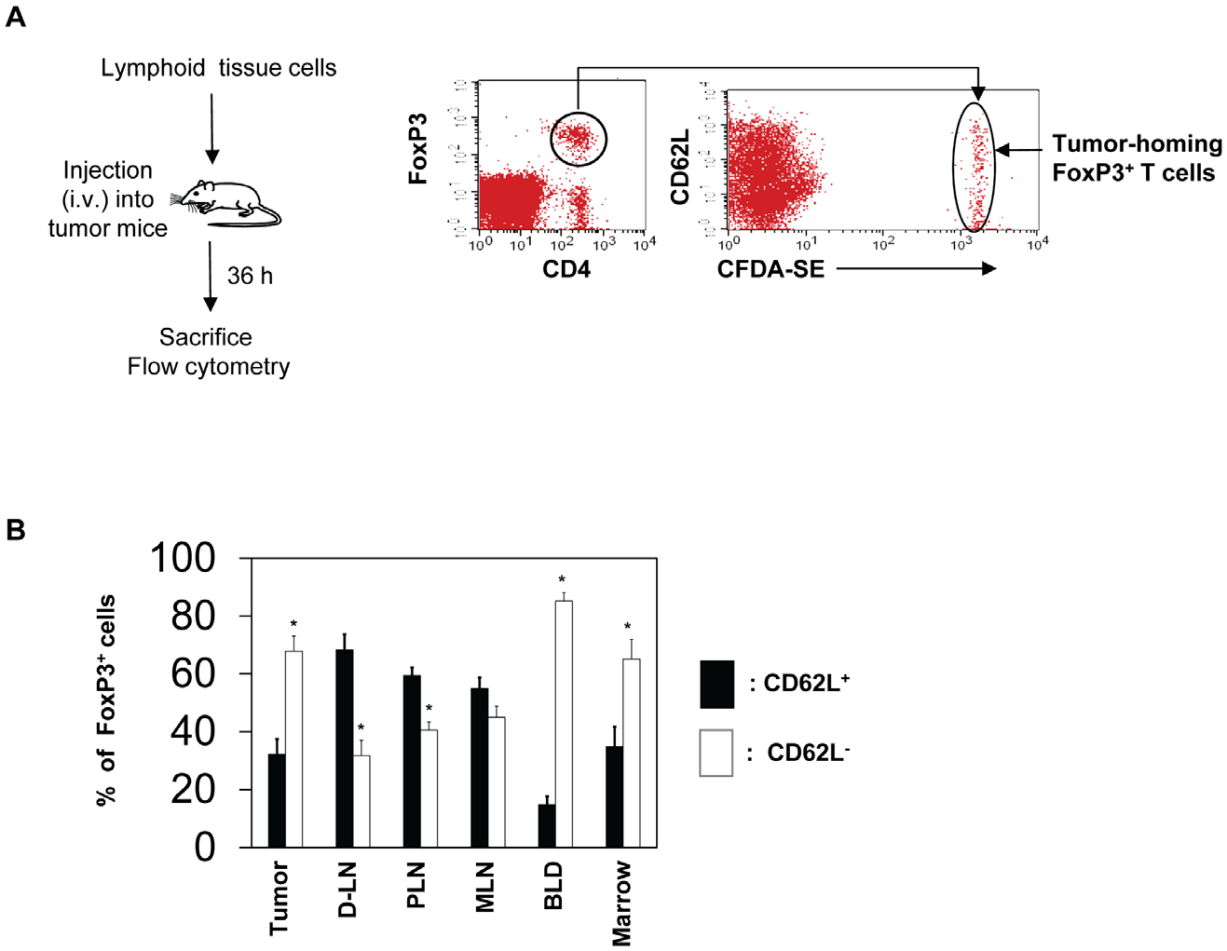
Memory type FoxP3^+^ regulatory T cells are more efficient than naïve type FoxP3^+^ T cells in migration into tumors. Pooled mononuclear cells including FoxP3^+^ T cells prepared from spleen, MLN and PLN were injected into BALB/c mice bearing A20 tumors, and the mice were sacrificed 36 hours later. (A) Detection of the migration of FoxP3^+^ cells into tumors. (B) Frequencies of naïve versus memory FoxP3^+^ T cells that migrated into various organs and tumors. Naïve FoxP3^+^ T cells are defined as CD62L^+^ (CD44^−^) cells, and memory FoxP3^+^ T cells are defined as CD62L^−^ (CD44^+/−^) cells. Representative and combined data obtained from 4 different experiments are shown. “*” indicates significant differences between frequencies of naïve and memory FoxP3^+^ T cells.

Direct migration of preformed FoxP3^+^ T cells into tumors is a potential mechanism for the enrichment of FoxP3^+^ T cells in tumors. We examined this possibility by performing a short term homing assay. FoxP3^+^ T cells were injected into A20 tumor-bearing mice and their migration into tumors within the 36 h time period was examined (Fig. 4A). On average, ∼10% of injected FoxP3^+^ T cells migrated into tumors among the organs examined, and this migration rate for tumors was comparable to other organs such as peripheral and mesenteric lymph nodes and significantly better than that of marrow (not shown). When the CD62L expression phenotype was examined, the majority of FoxP3^+^ T cells that migrated into tumors were CD62L-negative (Fig. 4B). This phenotype was similar to those migrated into the bone marrow or circulating in blood. In contrast, those migrated into the peripheral lymph node (PLN) and the mesenteric lymph node (MLN) were CD62L-positive. Thus, these results indicate that CD62L-negative memory type FoxP3^+^ T cells actively migrate into tumors.

### Antigen priming promotes both down-regulation of CCR7 and migration of FoxP3^+^ T cells into tumors

**Figure 5:**
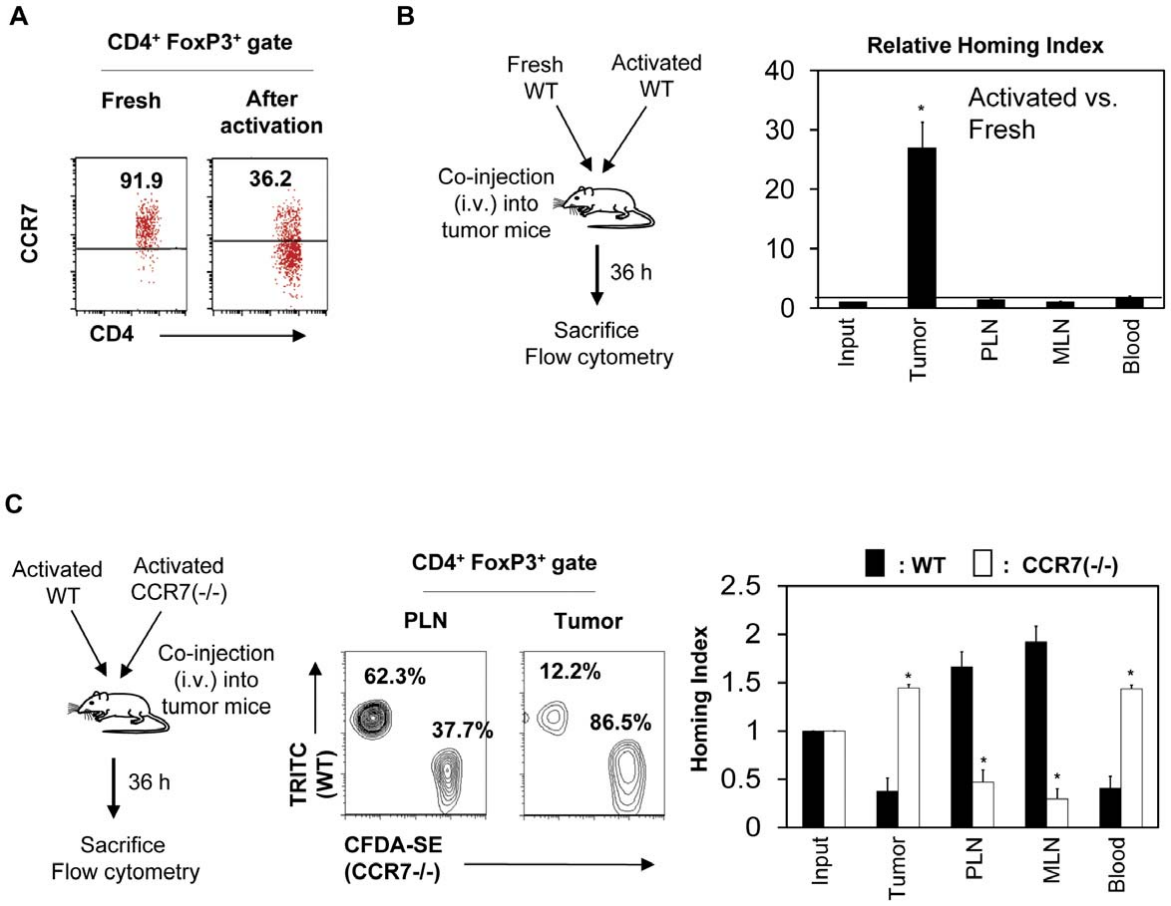
FoxP3^+^ cells lose CCR7 expression upon antigen priming, and only antigen primed CCR7^low^ cells can efficiently migrate into tumors. (A) Loss of CCR7 on FoxP3^+^ T cells following antigen priming. OVA-specific FoxP3^+^ T cells isolated from RIP-mOVA×DO11.10 Rag2 (−/−)mice were cultured in the presence of the OVA_323–339_ peptide and irradiated splenocytes as antigen presenting cells for 7 days, and CCR7 expression by FoxP3^+^ T cells was examined. (B) Comparison of the homing ability of antigen primed versus naïve FoxP3^+^ T cells. Fresh and antigen-primed FoxP3^+^ T cells were co-injected i.v. into 4T1-OVA tumor-bearing mice and the relative migration of the two FoxP3^+^ T cell populations into tumors and other organs were determined 36 h following the injection. (C) Comparison of antigen primed wild type and CCR7 (−/−) FoxP3^+^ T cells into tumors. CD4^+^ T cells, isolated from wild type and CCR7−/− mice were antigen primed in vitro for 5 days and injected into B16-tumor bearing mice. The relative migration of the two FoxP3^+^ T cell populations into tumors and other organs were determined 36 h following the injection. Representative and combined data obtained from 4–5 different experiments are shown. “*” indicates significant differences between the tumor and indicated organs (B) or between WT and CCR7 (−/−) FoxP3^+^ T cells (C).

The data presented so far implicate the loss of CCR7 and CD62L and memory phenotype in infiltration of tumors with FoxP3^+^ T cells. Because this phenotype is induced following antigen priming, we compared the expression of CCR7 and the migration ability of antigen primed and non-primed FoxP3^+^ T cells. We activated OVA-specific FoxP3^+^ T cells isolated from RIP-mOVA×DO11.10 Rag2 (−/−) mice with the OVA_323–339_ peptide and irradiated splenocytes as antigen presenting cells for 7 days in vitro. We comparatively examined the CCR7 expression and migration ability of the activated and freshly isolated lymphoid tissue FoxP3^+^ T cells. Antigen-primed FoxP3^+^ T cells were largely negative in expression of CCR7 (Fig. 5A) and were greatly enhanced in their ability to migrate into 4T1-OVA tumors compared to the freshly isolated lymphoid tissue FoxP3^+^ T cells (Fig. 5B).

The enhanced migration of antigen-primed FoxP3^+^ T cells could be due to acquisition of memory cell-associated non-lymphoid tissue homing receptors and/or loss of CCR7. To determine if loss of CCR7 plays any role in the migration, we utilized CCR7 (−/−) mice. We compared the migration of antigen primed CCR7 (−/−) and wild type CCR7 (+/+) cells into tumors. CCR7 (−/−) FoxP3^+^ T cells were not able to efficiently migrate into PLN and MLN but more efficiently migrated into tumors, compared to CCR7 (+/+) FoxP3^+^ T cells (Fig. 5C). The numbers of CCR7 (−/−) FoxP3^+^ T cells were greater than the numbers of their CCR7 (+/+) counterparts in the blood, suggesting that the cells that could not migrate into secondary lymphoid tissues were instead circulating in the blood.

**Figure 6:**
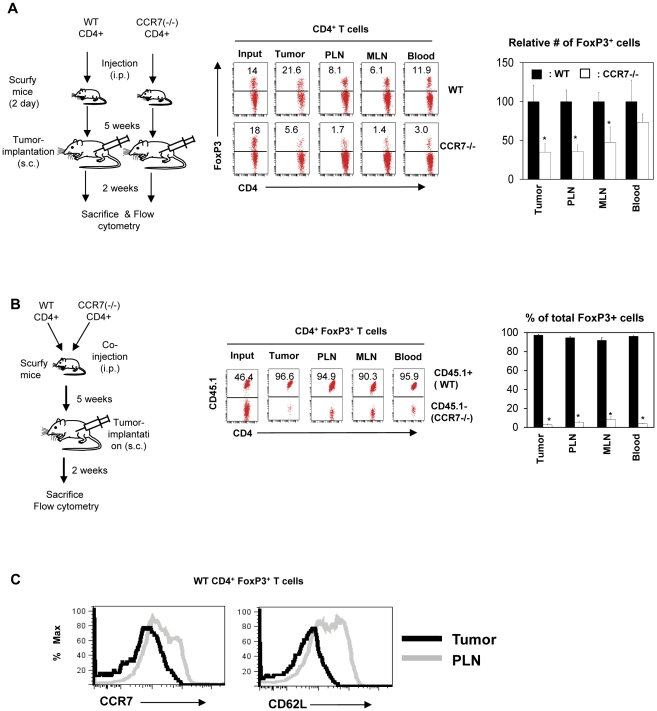
Naïve FoxP3^+^ T cells that cannot migrate first to lymphoid tissues fail to populate tumors. (A) Population of wild type versus CCR7 (−/−) FoxP3^+^ T cells in tumors. Wild type and CCR7 (−/−) CD4^+^ T cells, which were freshly isolated from lymphoid tissues, were separately transferred into scurfy mice on day 2 following birth. B16 tumor cells were implanted into the scurfy mice at 5 weeks of age, and the mice were sacrificed 2 weeks later. (B) Competitive population of wild type and CCR7 (−/−) FoxP3^+^ T cells in tumors. Wild type CD45.1^+^ CD4^+^ T cells and CCR7 (−/−) CD45.2^+^ CD4^+^ T cells were co-transferred into FoxP3-deficient scurfy mice on day 2 following birth. B16 tumor cells were implanted into the scurfy mice at 5 weeks of age, and the mice were sacrificed 2 weeks later. (C) Expression of CCR7 and CD62L by wild type FoxP3^+^ T cells populating tumors or lymph nodes of scurfy mice. Representative and combined data obtained from 3–4 different experiments are shown. “*” indicates significant differences between wild type and CCR7 (−/−) FoxP3^+^ T cells.

The data indicate the presence of a potential negative role for CCR7 in population of FoxP3^+^ T cells in tumors. The established function of CCR7 is to guide T cells into secondary lymphoid tissues for antigen priming. Without the migration and antigen priming in secondary lymphoid tissues, FoxP3^+^ T cells may not populate the tumors well. To determine the role of CCR7 in population of lymphoid tissues and then eventually into tumors, we compared the abilities of CCR7 (+/+) and CCR7 (−/−) FoxP3^+^ T cells separately transferred into tumor-bearing scurfy mice, which lack FoxP3^+^ T cells and develop systemic inflammation (Fig. 6A). CCR7 (−/−) FoxP3^+^ T cells were less efficient in population of lymph nodes and tumors. We also co-transferred CCR7 (+/+) and CCR7 (−/−) FoxP3^+^ T cells for competitive population in tumor-bearing scurfy mice (Fig. 6B). Most FoxP3^+^ T cells populating both lymphoid tissues and tumors were CCR7 (+/+), but not CCR7 (−/−), cells. Importantly, the wild type FoxP3^+^ T cells that migrated into tumors in scurfy mice were largely deficient in expression of CCR7 and CD62L (Fig. 6C). These results together with other data indicate that CCR7 is important for migration of FoxP3^+^ T cells into lymph nodes for antigen priming of FoxP3^+^ T cells. Antigen primed FoxP3^+^ T cells lose CCR7 which is important for migration and therefore population of FoxP3^+^ T cells in tumors.

### Decreased expression of CCR7 accompanies up-regulation of other trafficking receptors on tumor-infiltrating FoxP3^+^ T cells

**Figure 7:**
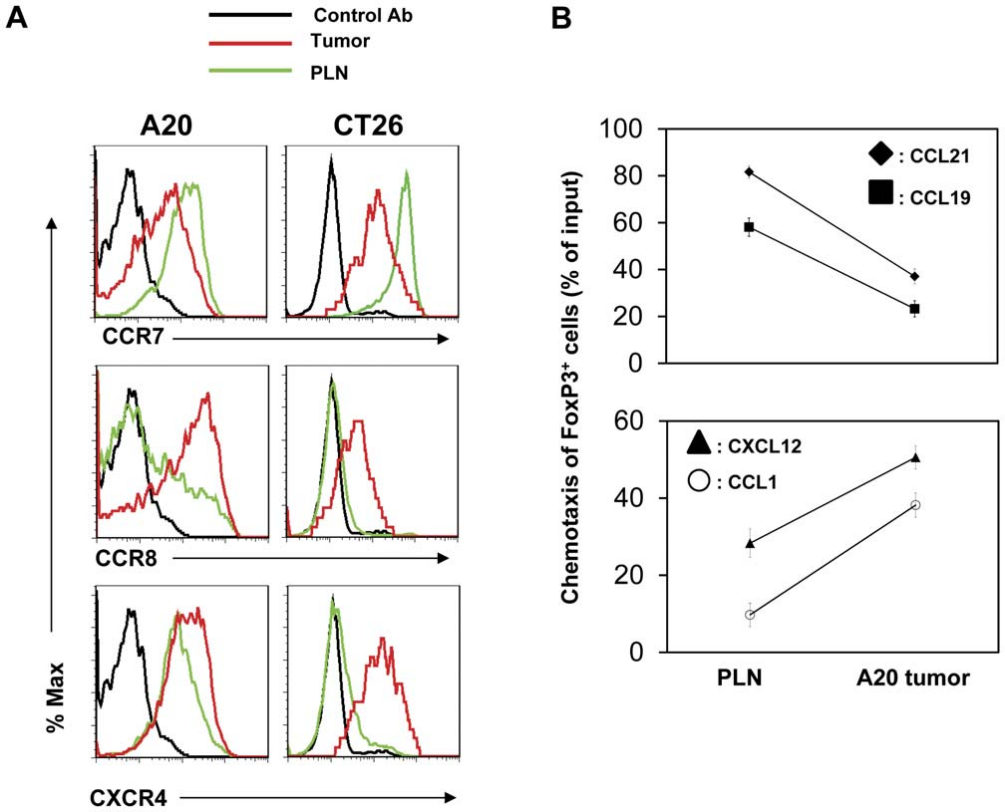
Expression of memory T cell trafficking receptors by tumor infiltrating FoxP3^+^ T cells. (A) Expression of CCR7, CCR8 and CXCR4 by tumor versus PLN-residing FoxP3^+^ T cells. (B) Chemotaxis of tumor versus PLN-residing FoxP3^+^ T cells to CCL19 and CCL21 (CCR7 ligands), CCL1 (CCR8), and CXCL12 (CXCR4). Cells from A20 tumor-bearing mice were examined. Representative (A) or combined (B) data obtained from 4–7 different experiments are shown.

It has been established that FoxP3^+^ T cells undergo a trafficking receptor switch following antigen priming in secondary lymphoid tissues. It is likely that a similar switch could occur for tumor-infiltrating FoxP3^+^ T cells. We examined expression of CCR8 and CXCR4, two chemokine receptors associated with migration of Tregs, by the FoxP3^+^ T cells in the lymph node and tumors. We observed that tumor-infiltrating FoxP3^+^ T cells highly expressed CCR8, while lymph node-residing FoxP3^+^ T cells express CCR8 at a relatively lower level. CXCR4, while expressed by FoxP3^+^ T cells of both tissues, was better expressed by tumor-infiltrating FoxP3^+^ T cells (Fig. 7A). We performed an in vitro chemotaxis assay with the chemokine ligands for CCR8 and CXCR4 (CCL1 and CXCL12) and observed these chemokines induced more efficient chemotaxis of tumor-infiltrating FoxP3^+^ T cells compared to their counterparts in lymph nodes (Fig. 7B). At the same time, the responses of the FoxP3^+^ T cells in tumors to CCR7 ligands (CCL19 and CCL21) were significantly decreased compared to their counterparts in lymph nodes. These results indicate the presence of a trafficking receptor switch from “CCR7^+^ CXCR4^medium^CCR8^−^” to “CCR7^−^ CXCR4^high^CCR8^+^” for migration of FoxP3^+^ T cells from the lymph node into tumors.

### Most FoxP3^+^ T cells in tumors are highly stable and retain FoxP3 expression

**Figure 8:**
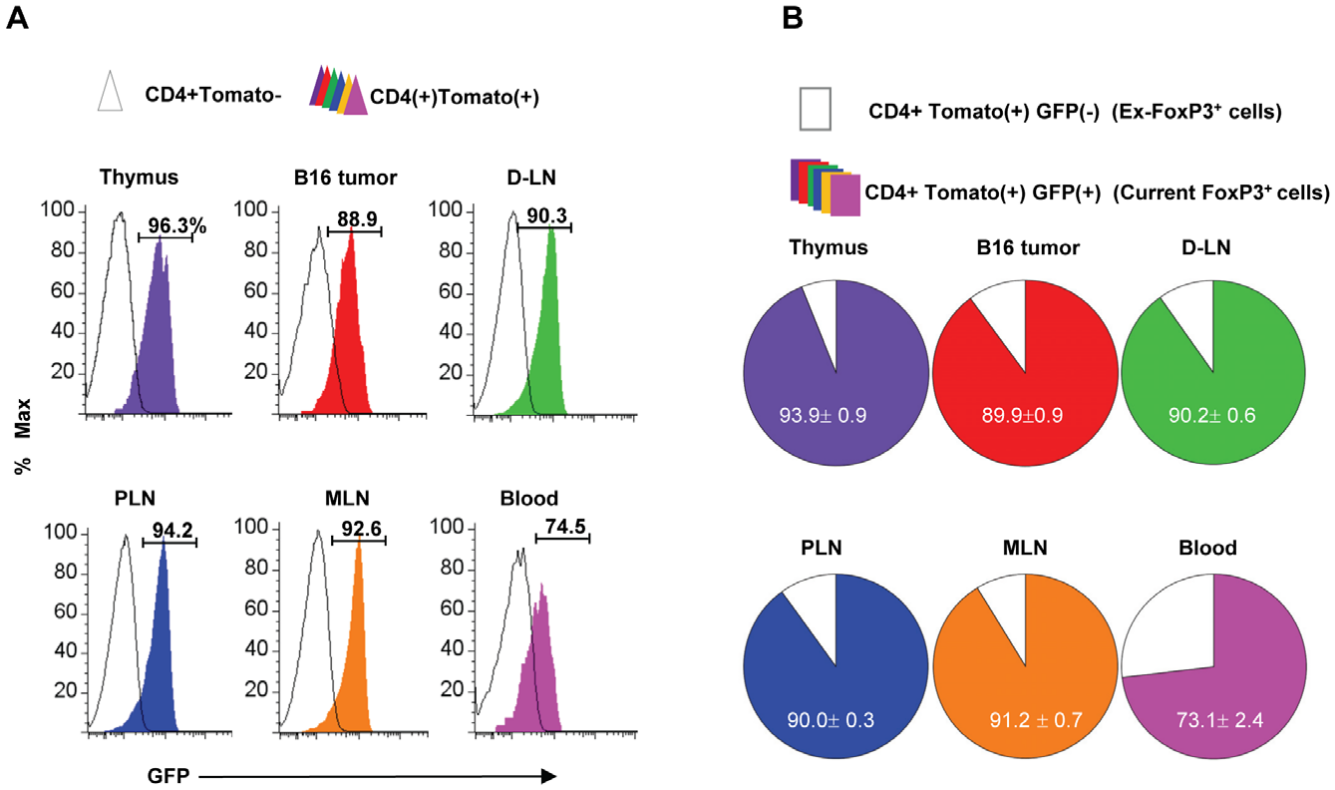
Most tumor-infiltrating FoxP3^+^ cells retain FoxP3 expression in vivo. FoxP3-GFP-Cre×Rosa-tdTomato mice were implanted with B16 cells to form tumors. Expression of GFP on tdTomato^+^ cells was examined by flow cytometry. GFP^+^ tdTomato^+^ T cells are current FoxP3^+^ T cells, whereas GFP^−^ tdTomato^+^ T cells are ex-FoxP3^+^ T cells. Representative (A) and combined (B) data obtained from 4 different experiments are shown.

Once migrated into tumors, FoxP3^+^ T cells are believed to undergo antigen priming for maintenance or expansion. It has not been determined if these FoxP3^+^ T cells would stay as FoxP3^+^ T cells or lose the FoxP3 expression and become FoxP3^−^ non-Tregs. We formed B16 tumors on the flank of FoxP3-GFP-Cre×Rosa-tdTomato mice. These mice express two fluorescent proteins GFP and tdTomato under the control of the FoxP3 promoter. Current FoxP3^+^ T cells are GFP^+^ tdTomato^+^, whereas ex-FoxP3^+^ T cells converted back to FoxP3^−^ cells are GFP^−^ tdTomato^+^. We found that the majority of tumor-infiltrating FoxP3^+^ T cells were GFP^+^ tdTomato^+^ cells (Fig. 8A and B). Only ∼10% of tumor-infiltrating FoxP3^+^ T cells were GFP^−^ tdTomato^+^ cells, suggesting that only ∼1 out 10 FoxP3^+^ T cells in tumors lost the FoxP3 expression since their immigration into tumors or generation.

## Discussion

Tregs, best exemplified by FoxP3^+^ T cells, have potent regulatory functions through expression of immune regulatory factors such as LAP-TGFß1, IL-10, and IL-35 [38]–[41]. Enrichment of Tregs in many types of tumors is believed to have significant impacts on anti-tumor immune responses and cancer patients [42]. Previous studies have indicated that migration of Tregs into tumors would play major roles in infiltration of Tregs in tumors [16], [24], [43]–[46]. Some groups reported that induced Tregs are the major cell type in tumors, and tumor-derived TGFß1 is important for induction of tumor-associated Tregs [27], [47], [48]. Despite the previous studies, the biological processes that regulate the enrichment of FoxP3^+^ T cells in tumors are still unclear. We performed experiments to provide insights into the biological processes that contribute to population of FoxP3^+^ T cells in tumors. The results indicate that Helios^+^ FoxP3^+^ T cells, antigen primed in secondary lymphoid tissues, are the major subset of Tregs in tumors. Additionally, the results provide insights into the important roles of antigen priming in draining lymph nodes and down-regulation of lymphoid tissue homing receptors and subsequent infiltration of FoxP3^+^ T cells into tumors. In addition, our study provides insights into the stability of FoxP3^+^ T cells in-tumors.

Prevailing theories to account for the enrichment of Tregs in tumors are 1) the induction theory [25]–[28] and 2) the migration theory [16]. Regarding the “induction theory,” it is believed that tumors produce certain factors that promote the conversion of conventional FoxP3^−^ T cells into FoxP3^+^ regulatory T cells. Factors that are known for generation of Tregs include cytokines and tissue factors including TGFβ1, retinoic acid, prostaglandin, progesterone and metabolic/micro-environmental factors [28], [49]–[56]. Most importantly, induction of FoxP3^+^ T cells requires TCR activation or antigen priming, which is required also for generation of all effector T cells. Because of the variability in availability of Treg-inducing tissue factors and antigens, tumors are expected to be extremely heterogeneous in their Treg-inducing capacity. Our data indicate that induction of Tregs in tumors would occur at low levels compared to induction in the tumor-draining lymph node. This is perhaps because induced Tregs are mostly generated from naïve T cells which are rare in the tumors that we examined in this study.

Helios is a member of the Ikaros transcription factor family and is preferentially expressed by thymus-derived FoxP3^+^ T cells but decreased in induced Tregs in mice [35]. Our data indicate that tumor-infiltrating FoxP3^+^ T cells are largely positive for Helios expression. In support of our observation, it has been recently reported that peripheral blood Tregs in human patients with renal cell carcinoma were mostly positive for Helios [57]. These observations may support the view that most tumor-infiltrating FoxP3^+^ T cells are derived originally from the FoxP3^+^ T cells generated in the thymus. This information, however, should be interpreted with caution if Helios expression is further regulated by non-thymus factors. Indeed, expression of Helios is transiently detected in activated T cells that are undergoing proliferation [36]. Therefore, high expression of Helios by tumor FoxP3^+^ T cells most likely indicates the activation state of the T cells in tumors.

Most tumor-associated FoxP3^+^ T cells are CD62L^−^ CCR7^−^. Antigen-primed CCR7 (−/−) FoxP3^+^ T cells were more efficient than antigen primed CCR7 (+/+) FoxP3^+^ T cells in migration into tumors. This indicates that loss of CCR7 has a direct impact on migration of Tregs into tumors. Paradoxically, our results indicate that primarily naïve antigen-inexperienced CCR7 (−/−) FoxP3^+^ T cells fail to populate tumors. This is because antigen priming is required for generation of tumor-infiltrating Tregs in addition to simple loss of CCR7, which normally occurs during differentiation of T cells in secondary lymphoid tissues. In this regard, non-antigen primed naïve FoxP3^+^ T cells are inefficient in migration into tumors. In addition to CCR7, down- or up-regulation of other trafficking receptors such as adhesion molecules (e.g. CD62L) and memory type trafficking receptors should occur for generation of Tregs that can efficiently migrate into tumors. Certain memory type trafficking receptors such as CCR4, CXCR4, and CCR5 are implicated in migration of FoxP3^+^ T cells into tumors [16], [23], [24], [58]. Our data indicate that CCR8 and CXCR4 are up-regulated on tumor-infiltrating FoxP3^+^ T cells. Loss of CCR7 and CD62L occurs during antigen priming in secondary lymphoid tissues and is important for migration of T cells to non-lymphoid tissues. CCR7 and CD62L are the most important trafficking receptors for migration of T cells into secondary lymphoid tissues such as the tumor-draining lymph node [59]. We observed that tumor-infiltrating FoxP3^+^ T cells highly express CCR8 and CXCR4, chemokine receptors associated respectively with memory cells and tumors. Therefore, loss of CD62L and CCR7 and up-regulation of certain memory type trafficking receptors are closely linked to migration of FoxP3^+^ T cells from secondary lymphoid tissues into tumors.

In the tumor types where Treg frequencies are linked to increased tumor growth and decreased patient survival, Treg depletion would be useful to mount anti-tumor immunity and enhance patient survival. Our results imply that simple depletion of the Tregs specifically in the tumors would not be effective as preformed Tregs can continually immigrate into tumors from the circulation. Therefore, it would be more effective to comprehensively deplete all Tregs that can migrate into tumors. In addition to depletion of Tregs, blocking the migration of Tregs into tumors is a potentially beneficial treatment. Blocking CCR8 and CXCR4 may lead to inhibition of migration of Tregs into tumors. A caveat is that some non-Treg effector T cells would also express these receptors [5] and their migration could be affected by the manipulation at the same time. In this case, anti-tumor immunity could be negatively affected by blocking of the trafficking receptors.

It is thought that tumors produce certain tissue factors that promote the induction and maintenance of Tregs. Our results underlie the importance of migration and maintenance of Tregs for their infiltration into tumors. In this regard, most T cells that were once FoxP3^+^ T cells retain their expression of FoxP3 in tumors, suggesting that tumor-infiltrating FoxP3^+^ T cells are highly stable in tumors. There are two reasons that make induction of FoxP3^+^ T cells in tumors inefficient. First, naïve FoxP3^−^ cells, which are preferential precursors of FoxP3^+^ T cells, would not readily migrate into tumors and therefore are rare in many types of tumors. Second, conventional memory FoxP3^−^ T cells, while they can become effector T cells, are relatively inefficient to become FoxP3^+^ T cells. Our results, however, do not rule out the possibility of induction of Tregs in tumors because Treg induction in tumors is likely to be dependent on the tumor type and anatomical location of tumors.
